# Lead optimization of AMS-17 identifies AMS-4 as a modulator of inflammasome-driven cocaine-associated neuroinflammation

**DOI:** 10.1016/j.bbrc.2026.153409

**Published:** 2026-02-05

**Authors:** Seema Singh, Naveen Kumar Gupta, Aitizaz UL Ahsan, Abiola Oladapo, Luis O. Mata, Shilpa Buch, Amol Kulkarni, Palsamy Periyasamy

**Affiliations:** a Department of Pharmacology and Experimental Neuroscience, University of Nebraska Medical Center, Omaha, NE, 68198, USA; b Department of Chemistry, University of Texas at El Paso, El Paso, TX, 79968, USA; c Department of Clinical Laboratory Sciences, University of Texas Medical Branch at Galveston, Galveston, TX, 77555, USA

**Keywords:** Microglia, Cocaine, NLRP3 inflammasome, Tertiary sulfonylurea derivatives

## Abstract

Cocaine use disorder (CUD) affects approximately 22.5 million people worldwide and is a major public health concern due to its widespread abuse and severe neuropsychiatric effects. Cocaine exposure induces neuroinflammation primarily through NLRP3 inflammasome activation in microglial cells, which releases proinflammatory cytokines such as IL-1β that contribute to neuronal dysfunction, making NLRP3 a key mediator of cocaine-induced neuropathology. Due to its critical role in neuroinflammatory signaling, the NLRP3 inflammasome is a promising therapeutic target for CUD. However, the development of effective and selective NLRP3 inhibitors remain limited due to challenges with specificity, bioavailability, and off-target effects. Thus, this study aimed to develop novel tertiary sulfonylurea compounds with selective inhibitory activity against the NLRP3 inflammasome and to investigate their efficacy in the context of cocaine exposure. We designed and synthesized five tertiary sulfonylurea-derived compounds. These compounds were structurally-inspired from AMS-17 by changing the two aromatic rings in AMS-17, while maintaining the cyclic urea. We tested their effects on NLRP3 activation in microglia. Thus, the BV2 cells were pre-treated with the novel compounds for 1 h, followed by cocaine exposure. Expression of NFκB, NLRP3 and its downstream signaling executer proteins were analyzed by Western blotting. Our results demonstrate that AMS-17 and its four derivatives exhibited varying levels of inhibition, with AMS-4 demonstrating the most pronounced effect, significantly reducing both NLRP3 and IL-1β expression. These findings suggest that selective sulfonylurea-based NLRP3 inhibitors, particularly AMS-4, may represent a promising therapeutic strategy for mitigating neuroinflammation associated with CUD.

## 1. Introduction

Cocaine remains one of the most commonly abused substances and is the second leading cause of drug overdose-related deaths in the United States [1]. Cocaine use has witnessed an alarming increase among young adults in recent years [2]. Cocaine Use Disorder (CUD) is associated with a wide range of neurological, psychiatric, and systemic complications, representing a significant burden on healthcare systems [3]. CUD is linked to numerous central nervous system pathologies, including an elevated risk of neurovascular disease [4], cognitive impairment [3], depression [3] and sudden cardiac death [1]. The rate of cocaine-related overdose deaths has surged dramatically – from 1.8 in 2003 to 8.9 in 2023 per 100,000 population, highlighting the urgent need for effective therapeutic strategies targeting CUD [5].

Cocaine activates microglia, promoting a proinflammatory phenotype that plays a central role in mediating cocaine-induced neurotoxicity [6]. Of the molecular pathways activated by cocaine, the NOD-, LRR- and pyrin domain-containing protein 3 (NLRP3) inflammasome has emerged as a key mediator of cocaine-induced neuroinflammation [7]. Cocaine functions as a danger-associated molecular pattern (priming or signal 1), triggering translocation of NFκB to the nucleus, which promotes transcription of NLRP3 inflammasome and its components [8] Cocaine is also a strong inducer of reactive oxygen species (ROS). These ROS activate NLRP3 signaling in microglia *in vitro* and *in vivo*. Cocaine induced ROS provides an activation (signal 2) for NLRP3. Thus, cocaine functions as a dual inducer of inflammasome activation release of proinflammatory cytokines, including IL-1β and IL-18 [9-12].

Emerging evidence highlights the NLRP3 inflammasome as a potential therapeutic target in cocaine-induced neuroinflammation. NLRP3 inhibition by small molecules, such as MCC950, is shown to display neuroprotective effects in cellular and animal models of CUD [9]. However, the long-term use of MCC950 in treating CUD is limited by concerns such as hepatotoxicity [13] and off-target binding to proteins like carbonic anhydrase-2 [14]. These concerns underscore the need for safer, more effective, and selective NLRP3 inflammasome inhibitors for effective CUD therapy.

Structurally diverse natural products have shown NLRP3 inhibitory activity [15], but issues such as pleiotropic effects [16], and weak *in vitro* and *in vivo* correlation [17] limit their potential as drug leads. We previously identified and characterized AMS-17, a novel small-molecule inhibitor of the NLRP3 inflammasome, structurally inspired from the natural flavonoid isoliquiritigenin [18]. This study highlights the therapeutic potential of a tertiary sulfonylurea derivative AMS-17 and its analogues in attenuating cocaine-induced neuroinflammation.

## 2. Materials and methods

### 2.1. General information

All reactions were conducted under an argon atmosphere unless otherwise noted. The glassware for these reactions was dried in 120 °C overnight and cooled under argon atmosphere before running the reaction. The solvents and other chemicals/reagents were used as received, without any further purification. Column chromatography was carried out on Merck silica gel 60 (230 – 400 mesh). ^1^H NMR spectra were recorded at 400 MHz and ^13^C NMR spectra at 100 MHz in DMSO-*d*_6_. ^1^H NMR spectra were referenced on the DMSO-*d*_6_ signal at 2.5 ppm. The ^13^C NMR spectra were referenced at 39.50 ppm for DMSO-*d*_6_.

### 2.2. General procedure for synthesis of acyclic urea derivatives

A 10 mL round-bottom flask was charged with arylamine (0.5 mmol, 1.0 equiv.) and dichloromethane (CH_2_Cl_2_, 4 mL). 3-chloropropyl isocyanate (0.55 mmol, 1.1 equiv.) was added under argon atmosphere. The reaction mixture was stirred at an ambient temperature for 72 h and was monitored by thin layer chromatography. Upon completion of the reaction, the volatiles were removed under reduced pressure, and the resultant crude product was purified by silica gel column chromatography. Elution with appropriate solvent mixture (ethyl acetate (EtOAc) and hexanes resulted in the isolation of the desired acyclic urea as solid. Because of the stability issues with the acyclic sulfonylureas, they were subjected to the next step after confirming the structural identity by ^1^H- and ^13^C NMR.

### 2.3. 1-(3-Chloropropyl)-3-(pyrimidin-5-yl)urea (5)

**Yield** = 105 mg, 92% **R_*f*_** = 0.1 (EtOAc mobile phase), **Appearance** = light yellow solid, **^1^H NMR (400 MHz, DMSO-d_6_): δ** 8.83 (S, 3H, NH proton merged), 8.73 (s, 1H), 6.60 (t, *J* = 5.6 Hz, 1H), 3.67 (t, *J* =6.4 Hz, 2H), 3.23 (q, *J* =6.4 Hz, 2H), 1.90 (quint, *J* =6.8 Hz, 2H) ppm, **^13^C NMR (100 MHz, DMSO-d_6_): δ** 154.9, 151.3, 145.8, 135.7, 42.9, 36.8, 32.5 ppm.

**1-(3-Chloropropyl)-3-(1-methyl-1H-indol-5-yl)urea (6) Yield** = 110 mg, 83%, **R_*f*_** = 0.3 (50 % EtOAc and Hexane mobile phase), **Appearance** = Light brown, **^1^H NMR (400 MHz, DMSO-d6): δ** 8.18 (s, 1H), 7.61 (s, 1H), 7.27 (d, *J* = 8.8 Hz, 1H), 7.22 (d, *J* = 3.2 Hz, 1H), 7.07 (d, *J* = 8.8 Hz, 1H), 6.29 (d, *J* = 3.2 Hz, 1H), 6.13 (t, *J* = 5.4 Hz, 1H), 3.73 (s, 3H), 3.68 (t, *J* = 6.6 Hz, 2H), 3.21 (q, *J* = 9.2 Hz, 2H), 1.89 (quint., *J* = 6.6 Hz, 2H) ppm. **^13^C NMR (100 MHz, DMSO-d6): δ** 155.9, 132.6, 132.5, 129.8, 128.1, 114.5, 109.6, 109.4, 99.9, 43.1, 36.6, 32.9, 32.5 ppm.

### 2.4. 1-(Benzo[d]oxazol-6-yl)-3-(3-chloropropyl)urea (7)

**Yield** = 97 mg, 72%, **R_*f*_** = 0.3 (50% EtOAc and Hexane mobile phase), **Appearance** = Magenta fluffy solid, **^1^H NMR (400 MHz, DMSO-d6): δ** 8.75 (s, 1H), 8.55 (s, 1H), 8.04 (d, *J* = 2.0 Hz, 1H), 7.61 (d, *J* = 8.4 Hz, 1H), 7.16 (dd, *J* = 2.0, 8.4 Hz, 1H), 6.32 (t, *J* = 5.6 Hz, 1H), 3.68 (t, *J* = 6.4 Hz, 2H), 3.23 (q, *J* = 6.4 Hz, 2H), 1.90 (quint, *J* = 6.4 Hz, 2H) ppm. **^13^C NMR (100 MHz, DMSO-d6): δ** 155.3, 152.9, 149.9, 138.8, 133.6, 119.7, 115.3, 99.8, 43.0, 36.6, 32.6 ppm.

### 2.5. General procedure for the synthesis of cyclic urea derivatives

A round-bottom flask (25 mL capacity) was charged with acyclic urea (0.5 mmol, 1 equiv.) and anhydrous tetrahydrofuran (4 mL). The reaction mixture was cooled to 0 °C for 15 min using an ice bath. NaH (120 mg, 3.0 equivalent, 60% suspension in paraffin oil) was added slowly to the reaction mixture. The reaction mixture was stirred at 0 °C for 30 min, slowly warmed to the ambient temperature, and allowed to stir at an ambient temperature for 16 h. Appropriate arylsulfonyl chloride (0.55 mmol, 1.1 equivalent). The reaction was stirred at ambient temperature for the next 2 h. The crude was purified by silica gel column chromatography without further workup. Elution with EtOAc/hexanes afforded the corresponding cyclic urea derivatives.

### 2.6. 1-(Pyrimidin-5-yl)-3-((4-(trifluoromethyl)phenyl)sulfonyl) tetrahydropyrimidin-2(1H)-one (AMS-17)

**Yield** = 146 mg, 76% **R_*f*_** = 0.3 (EtOAc mobile phase), **Appearance** = colorless solid, **^1^H NMR (400 MHz, DMSO-d_6_): δ** 8.99 (s, 1H), 8.76 (s, 2H), 8.18 (d, *J* = 8.4 Hz, 2H), 7.98 (d, *J* = 8.4 Hz, 2H), 4.10 (t, *J* = 5.8 Hz, 2H), 3.79 (t, *J* = 5.8 Hz, 2H), 2.22 (quint, *J* = 5.6 Hz, 2H) ppm, **^13^C NMR (100 MHz, DMSO-d_6_): δ** 155.2, 153.7, 150.4, 143.5, 137.2, 132.9(q), 128.9, 127.4, 126.3, 126.1(q), 124.7, 122.0, 119.3, 48.1, 45.8, 22.0 ppm, **^19^F NMR (376 MHz, DMSO-d_6_): δ** −61.74 ppm. **HRMS (ESI, Q-ToF) m/z:** [M+Na]^+^ calculated for C_15_H_13_F_3_N_4_O_3_S 409.0558; Found 409.0557. IR (neat): 2918, 2847, 1673, 1656, 1558, 1474, 1427, 1405, 1360, 1321, 1287, 1164, 1140, 1103, 1060, 1030, 1006, 892, 842, 806, 743, 715 cm^−1^.

### 2.7. 1-(1-Methyl-1H-indol-5-yl)-3-((4-(trifluoromethyl)phenyl)sulfonyl) tetrahydropyrimidin-2(1H)-one (AMS-4)

**Yield** = 181 mg, 83% **R_*f*_** = 0.9 (0.1% MeOH and EtOAc mobile phase), **Appearance** = colorless solid, **^1^H NMR (400 MHz, DMSO-d6):** *δ* 8.15 (d, *J* = 8.4 Hz, 2H), 7.96 (d, *J* = 8.4 Hz, 2H), 7.38-7.33 (m, 3H), 6.95 (dd, *J* = 2.0, 8.4 Hz, 1H), 6.37 (dd, *J* = 0.6, 3.0 Hz, 1H), 4.09 (t, *J* = 5.8 Hz, 2H), 3.76 (s, 3H), 3.67 (t, *J* = 5.8 Hz, 2H), 2.19 (quint, *J* = 5.8 Hz, 2H) ppm, **^13^C NMR (100 MHz, DMSO-d6): δ** 150.5, 144.3, 134.9, 134.2, 132.7(q), 130.6, 127.9, 125.9(q), 124.8, 122.1, 120.0, 118.2, 109.8, 100.4, 50.0, 45.6, 32.6, 22.6 ppm, **^19^F NMR (376 MHz, DMSO-d_6_): δ** −61.62 ppm. **HRMS (ESI, Q-ToF) m/z:** [M+Na]^+^ calculated for C_20_H_18_F_3_N_3_O_3_S 460.0919; Found 460.0919. **IR (neat):** 2939, 1671, 1490, 1476, 1425, 1403, 1351, 1321, 1304, 1280, 1161, 1121, 1103, 1060, 1038, 1011, 877, 844, 810, 741, 715 cm^−1^.

### 2.8. 1-(Benzo[d]oxazol-6-yl)-3-((4-(trifluoromethyl)phenyl)sulfonyl) tetrahydropyrimidin-2(1H)-one (AMS-6)

**Yield** = 142 mg, 67% **R_*f*_** = 0.2 (90% EtOAc and Hexane mobile phase), **Appearance** = Dark Brown solid, **^1^H NMR (400 MHz, DMSO-d_6_): δ** 8.13 (d, *J* = 8.0 Hz, 2H), 7.96 (d, *J* = 8.0 Hz, 2H), 7.37-7.32 (m, 3H), 6.94 (dd, *J* = 2.2, 8.6 Hz, 1H), 4.08 (t, *J* = 5.6 Hz, 2H), 3.78 (s, 3H), 3.66 (t, *J* = 5.6 Hz, 2H), 2.19 (quint, *J* = 5.6 Hz, 2H) ppm, **^13^C NMR (100 MHz, DMSO-d_6_): δ** 150.5, 144.6, 134.9, 134.2, 132.7(q), 130.6, 131.2, 128.6, 127.9, 126.9, 126.0(q), 122.0, 118.2, 109.8, 50.1, 45.6, 32.5 ppm, **^19^F NMR (376 MHz, DMSO-d_6_): δ** −61.67 ppm. HRMS (ESI, Q-ToF) *m/z*: [M+Na]^+^ calculated for C_18_H_14_F_3_N_3_O_4_S 448.0555; Found 448.0545. **IR (neat):** 2953, 2924, 2853, 1664, 1476, 1427, 1403, 1375, 1351, 1319, 1300, 1164, 1125, 1107, 1080, 1013, 879, 834, 799, 765, 719 cm^−1^.

### 2.9. 1-(Pyridin-3-ylsulfonyl)-3-(pyrimidin-5-yl)tetrahydropyrimidin-2 (1H)-one (AMS-7)

**Yield** = 126 mg, 79% **R_*f*_** = 0.2 (20% MeOH and EtOAc mobile phase), **Appearance** = White solid, **^1^H NMR (400 MHz, DMSO-d_6_): δ** 9.09 (s, 1H), 8.99 (s, 1H), 8.84 (d, *J* = 4.0 Hz, 1H), 8.75 (s, 2H), 8.35 (d, *J* = 8.0 Hz, 1H), 7.66-7.63 (m, 1H), 4.09 (t, *J* = 5.6 Hz, 2H), 3.77 (t, *J* = 5.6 Hz, 2H), 2.20 (t, *J* = 5.6 Hz, 2H)ppm, **^13^C NMR (100 MHz, DMSO-d_6_): δ** 155.2, 153.8, 153.7, 150.5, 148.3, 137.2, 136.2, 136.2, 123.9, 48.1, 45.7, 21.9 ppm. **HRMS (ESI, Q-ToF) m/z:** [M+Na]^+^ calculated for C_13_H_13_N_5_O_3_S 342.0637; Found 342.0637. **IR (neat):** 1654, 1561, 1479, 1425, 1347, 1287, 1196, 1162, 1095, 1032, 887, 803, 749 cm^−1^.

### 2.10. 1-(Pyrimidin-5-yl)-3-(thiophen-2-ylsulfonyl)tetrahydropyrimidin-2 (1H)-one (AMS-8)

**Yield** = 123 mg, 76% **R_*f*_** = 0.2 (EtOAc mobile phase), **Appearance** = Light yellow solid, **^1^H NMR (400 MHz, DMSO-d_6_): δ** 9.01 (s, 1H), 8.79 (s, 2H), 8.04 (dd, *J* = 1.4, 5.0 Hz, 1H), 7.84 (dd, *J* = 1.4, 3.8 Hz, 1H), 7.20-7.18 (m, 1H), 3.98 (t, *J* = 6.0 Hz, 2H), 3.76 (t, *J* = 6.0 Hz, 2H), 2.17 (t, *J* = 6.0 Hz, 2H)ppm, **^13^C NMR (100 MHz, DMSO-d_6_): δ** 155.2, 153.7, 150.5, 138.9, 137.4, 135.0, 134.6, 127.3, 48.1, 45.7, 22.0 ppm. **HRMS (ESI, Q-ToF) m/z:** [M+Na]^+^ calculated for C_12_H_12_N_2_O_3_S_2_ 347.0249; Found 347.0249. **IR (neat):** 2927, 1669, 156, 1476, 1433, 1349, 1310, 1295, 1157, 1088, 1030, 892, 860, 799, 739 cm^−1^.

### 2.11. BV2 cell culture

The mouse microglial BV2 cell line, kindly provided by Dr. Sanjay Maggirwar (George Washington School of Medicine and Health Sciences, MD, USA), was maintained in Dulbecco's Modified Eagle's Medium (DMEM; Corning, Cat No. MT10013CV) containing 10% heat-inactivated fetal bovine serum (FBS; Thermo Fisher Scientific, Cat No. 16000-044) and 1% penicillin-streptomycin (10,000 U/mL penicillin and 10,000 μg/mL streptomycin; Life Technologies, Cat No. 15140-122). For experiments, BV2 cells were plated at a density of 0.2 × 10^6^ cells/well in 6-well plates. Cells were cultured at 37 °C in a humidified atmosphere containing 5% CO_2_. After 24 h, when they reached approximately 70% confluence, the cells were serum-starved for 8 h and then pretreated with AMS analogues (dissolved in DMSO) following cocaine exposure.

### 2.12. Drug preparation

AMS analogues were dissolved in DMSO to generate stock solutions (3-50 mM) and diluted in culture medium to final concentrations of 3-50 μM by adding 2 μL of stock to 2 mL DMEM; AMS-17 was prepared as a 30 mM DMSO stock and used at a final concentration of 30 μM. Cocaine was prepared as a 10 mM stock in 1x PBS and diluted to a final concentration of 10 μM by adding 2 μL to 2 mL DMEM.

### 2.13. Western blotting

At the end of experiments, BV2 cells were washed with warm PBS, lysed in RIPA buffer containing protease and phosphatase inhibitors, sonicated, centrifuged to collect supernatants, and total protein was quantified by BCA assay (Thermo Fisher Scientific, Cat No.23227) before denaturation in Laemmli buffer for Western blot analysis. Equal amounts of protein were separated by SDS-PAGE and transferred to PVDF membranes (Millipore Sigma, Cat. No. IPVH00010). Membranes were blocked with 5% nonfat dry milk for 1 h and incubated overnight at 4 °C with primary antibodies against NLRP3 (Abcam, Cat. No. AG-20B-0014-C; 1:3000) and IL-1β (Abcam, Cat. No. AB9722; 1:1000). After washing, membranes were incubated with HRP-conjugated secondary antibodies. Bands were visualized using SuperSignal substrate (Thermo Fisher Scientific, Cat. No. 34578) and imaged on a Fluorchem M imaging system (Cell Biosciences). β-Actin served as the loading control, and band intensities were quantified using ImageJ software [19].

### 2.14. qPCR

Following completion of the treatments, total RNA was isolated from BV2 cells using the Quick-RNA^™^ Miniprep Kit (Zymo Research Corporation, USA; Cat No. R1055) in accordance with the manufacturer's protocol. Subsequently, 1 μg of total RNA was reverse transcribed into cDNA using the Verso cDNA Synthesis Kit (Thermo Fisher Scientific, USA; Cat No. AB1453/B). Quantitative real-time PCR was carried out using TaqMan Universal Master Mix (Applied Biosystems) with probes targeting IL-1β (assay ID: Mm00434228), The expression of target genes was normalized to GAPDH (assay ID: Mm99999915) and relative mRNA expression levels were determined using the 2^−ΔΔCT^ method.

### 2.15. Statistical analysis

All experiments were performed with 3-4 biological replicates. Data are presented as the mean ± SEM. Statistical analyses were carried out using GraphPad Prism software (version 10.4.1; San Diego, CA, USA). One-way ANOVA, followed by Dunnett's multiple comparisons test, was employed for comparisons among multiple groups. Statistical significance was defined as a p-value below 0.05.

## 3. Results

### 3.1. Chemical synthesis of AMS-17 and its analogues

**Fig. 1.**
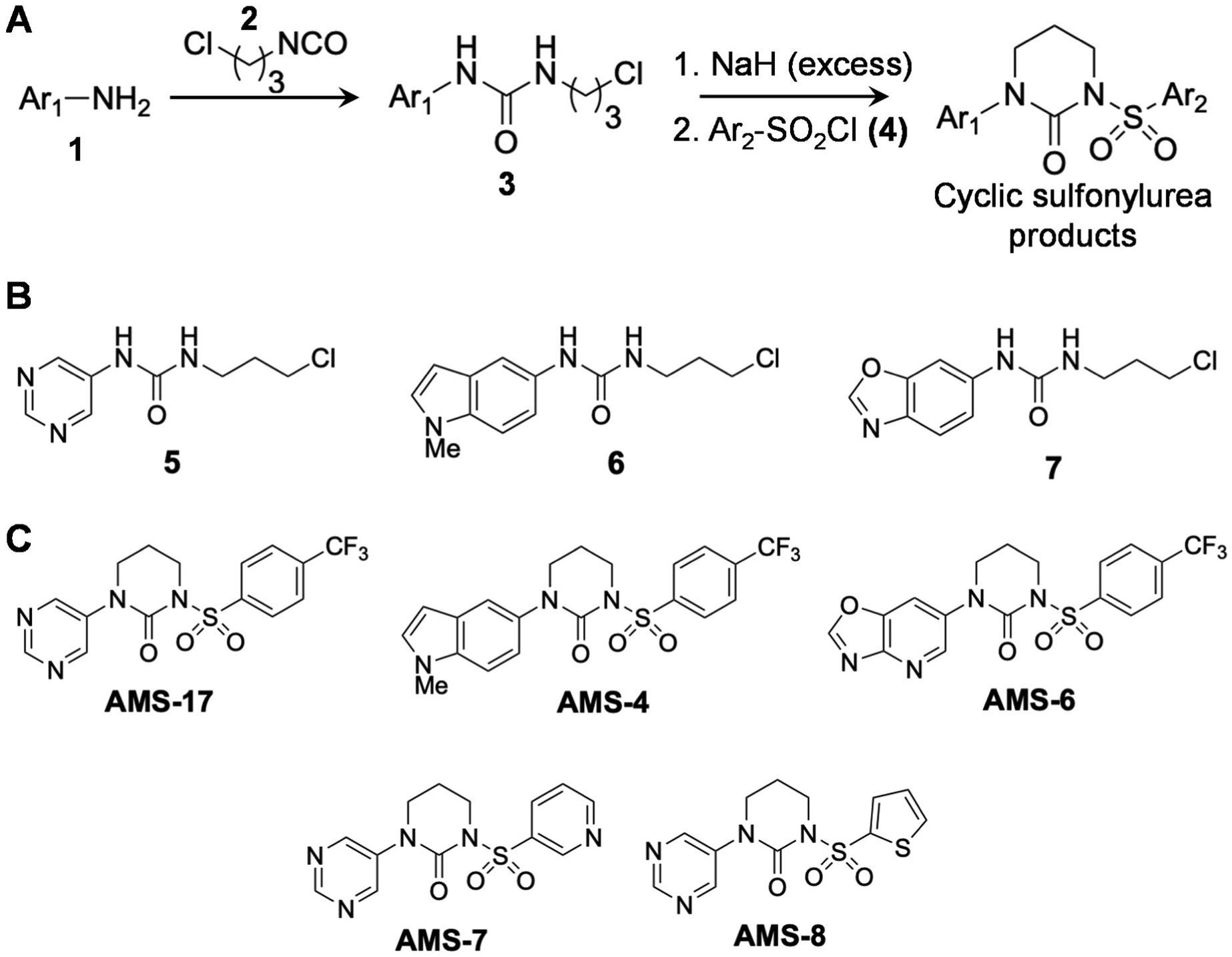
Structures of AMS-17 and their analogues A. General scheme for the chemical synthesis of sulfonylurea compounds. B. The structures of acyclic urea intermediates **5–7**. C. Structures of the cyclic urea compounds screened for the NLRP3 inhibitory activity.

The chemical synthesis of AMS-17 and the analogous tertiary sulfonylureas is highlighted in Fig. 1A. It is a slight modification of our previously reported protocol [18]. It began with the reaction of the corresponding arylamine (**1**) with 3-chloropropyl isocyanate (**2**) resulting in the formation of acyclic urea (**3**). Intramolecular cyclization mediated by sodium hydride followed by quenching the reaction with the appropriate sulfonyl chloride (**4**) resulted in the formation of the desired cyclic sulfonylurea products. The compounds were isolated, purified using flash column chromatography, and characterized using chromatographic and spectroscopic techniques. Fig. 1B represents the chemical structure of each AMS analogue and Fig. 1C represents the structures of the cyclic urea compounds screened for the NLRP3 inhibitory activity.

### 3.2. Effect of AMS-17 on cocaine-mediated NLRP3 inflammasome activation in BV2 cells

**Fig. 2.**
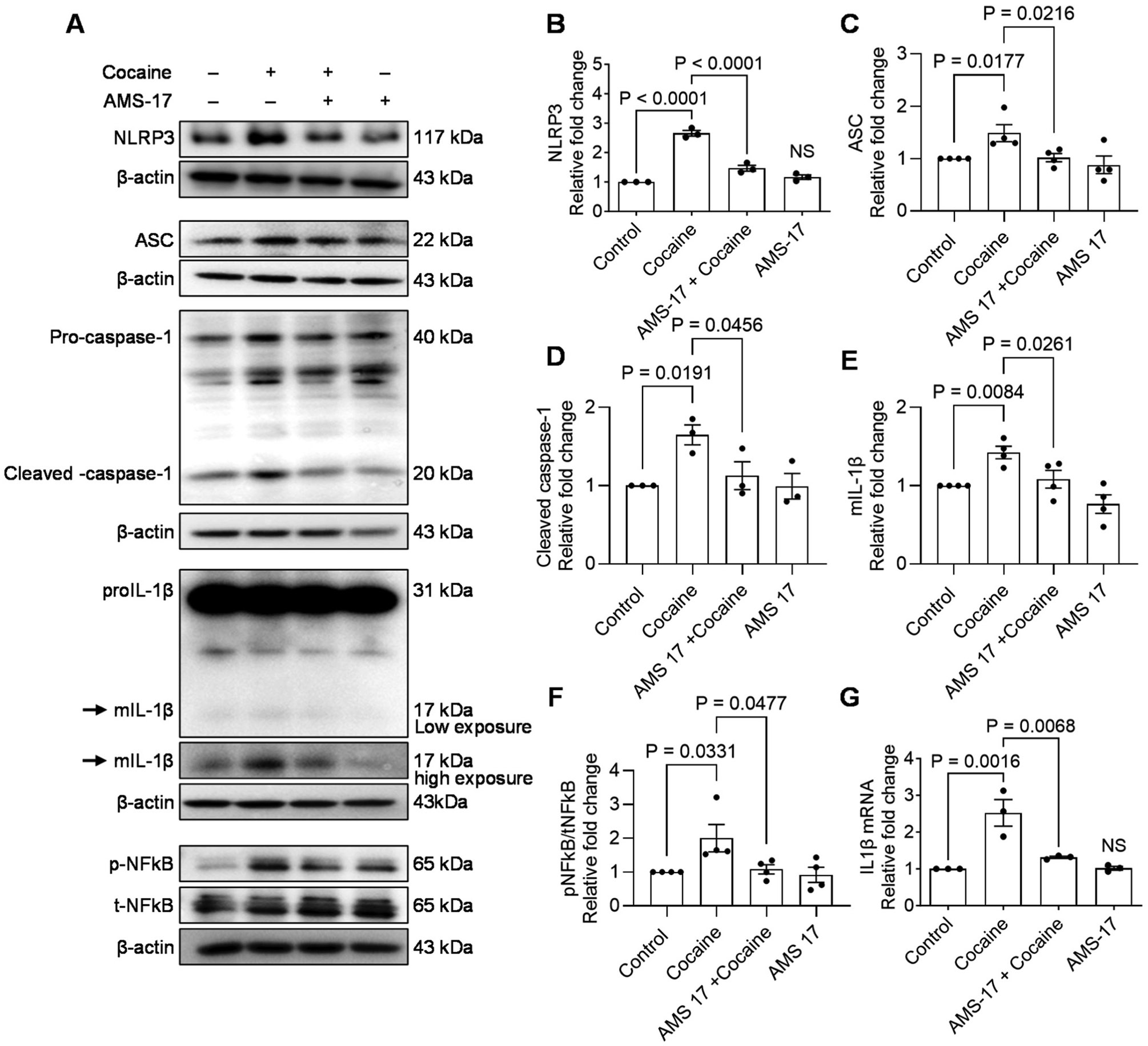
AMS-17 inhibits the NLRP3 signaling in cocaine stimulated BV2 cells. (A) Representative western blots showing protein expression of NLRP3, ASC, cleaved caspase-1, mature IL-1β, and phospho-NFκB in BV2 cells pretreated with AMS-17 (1 h) followed by cocaine exposure (24 h). β-Actin was used as a loading control. (B–F) Densitometric quantification of NLRP3, ASC, cleaved caspase-1, mature IL-1β, and phospho–NF–κB protein expression normalized to β-actin. (G) qPCR analysis of *Il1β* mRNA expression in BV2 cells under the same experimental conditions, with *Gapdh* used as the housekeeping gene. Data are presented as mean ± SEM; N = 3-4.

To evaluate the anti-inflammatory potential of AMS analogues, we first examined the effect of AMS-17 on cocaine-induced NLRP3 inflammasome signaling in BV2 microglial cells. Cells were pretreated with AMS-17 (30 μM) for 1 h prior to exposure to cocaine (10 μM) for 24 h. Protein levels of NLRP3 inflammasome components (NLRP3, ASC, and cleaved caspase-1), and mature IL-1β, were assessed by western blotting. AMS-17 pretreatment significantly attenuated cocaine-induced upregulation of NLRP3 expression (Fig. 2A and B) and reduced ASC and cleaved caspase-1 levels (Fig. 2A, C, and 2D), accompanied by a marked decrease in mature IL-1β production (Fig. 2A and E). To determine whether AMS-17 modulates inflammasome priming, we assessed NFκB activation and pro-inflammatory cytokine, *Il1β*, gene expression. AMS-17 significantly reduced cocaine-induced NFκB phosphorylation (Fig. 2A and F) and downregulated *Il1β* mRNA expression (Fig. 2G), indicating inhibition of the priming phase (signal 1). Together, these findings demonstrate that AMS-17 suppresses cocaine-induced NLRP3 inflammasome activation by inhibiting both NFκB-dependent priming and downstream inflammasome signaling in microglial cells.

### 3.3. Screening of AMS-compounds in cocaine-mediated activation of NLRP3 in BV2 cells

**Fig. 3.**
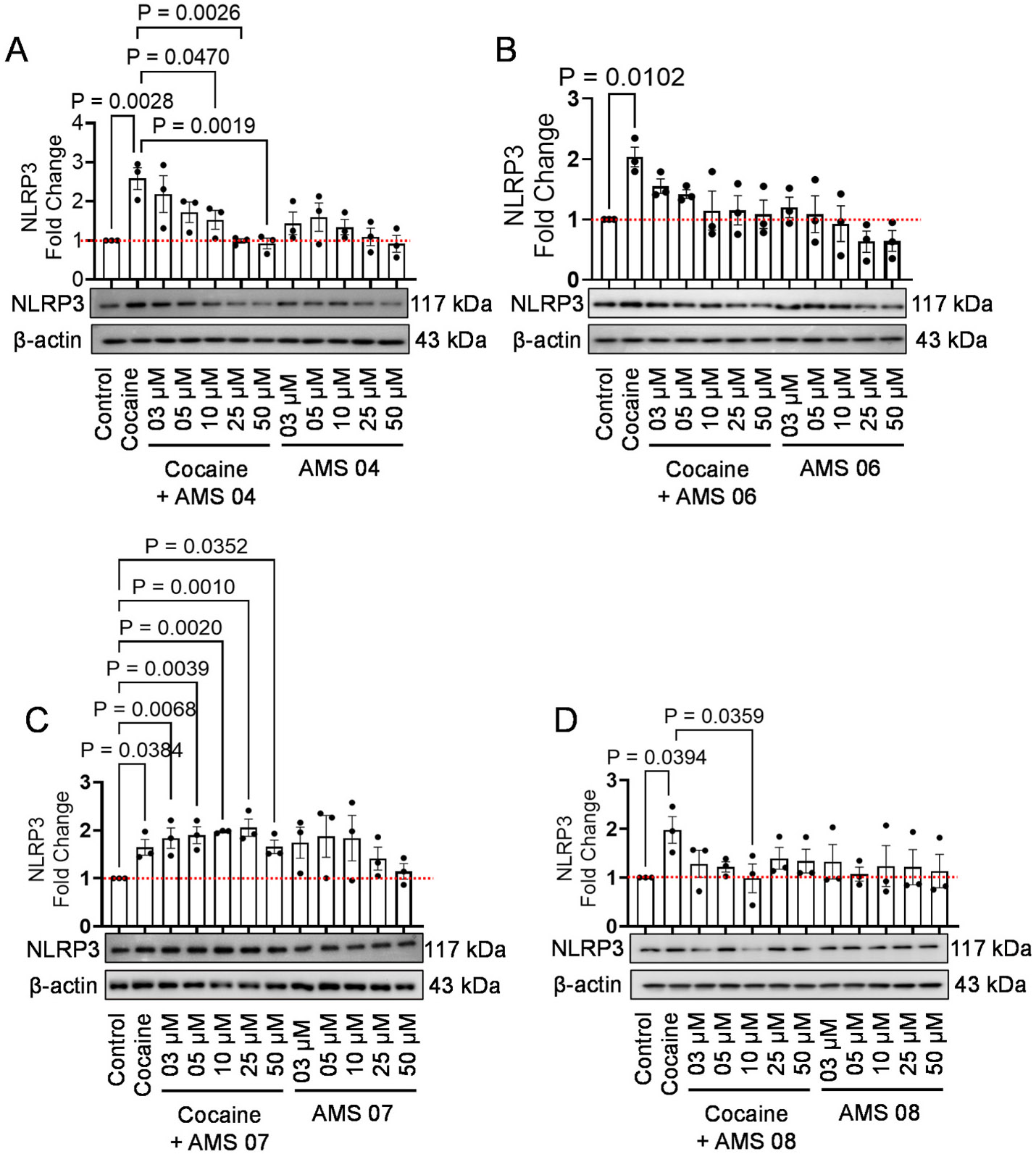
Screening of AMS-analogues against NLRP3 activity (inhibition) in cocaine stimulated BV2 cells. (A-D) Representative Western blot and corresponding bar graphs showing expression of NLRP3 in BV2 cells pre-exposed to AMS-4 (A), AMS-6 (B), AMS-7 (C), and AMS-8 (D) following 1 h cocaine (10 μM). β-actin was used as a loading control. Data are presented as mean ± SEM; N = 3.

Given the need to identify small molecules that selectively target NLRP3 inflammasome activation independent of upstream NFκB signaling, we next screened a panel of AMS analogues (AMS-4, AMS-6, AMS-7, and AMS-8) for their ability to suppress cocaine-induced NLRP3 activation in BV2 microglial cells. Cells were pretreated with increasing concentrations (3-50 μM) of each compound for 1 h prior to cocaine exposure, and NLRP3 protein expression was assessed. As shown in Fig. 3A-D, all screened AMS analogues, with the exception of AMS-7, significantly attenuated cocaine-induced NLRP3 expression. Among these, AMS-4 (Fig. 3A) exhibited the most pronounced inhibitory effect. In contrast, AMS-7 (Fig. 3C) failed to suppress NLRP3 expression across the tested concentrations. AMS-1 was excluded from further analysis due to poor solubility in DMSO, which precluded reliable evaluation.

### 3.4. AMS-4 attenuates cocaine-mediated downstream NLRP3 signaling in BV2 cells

**Fig. 4.**
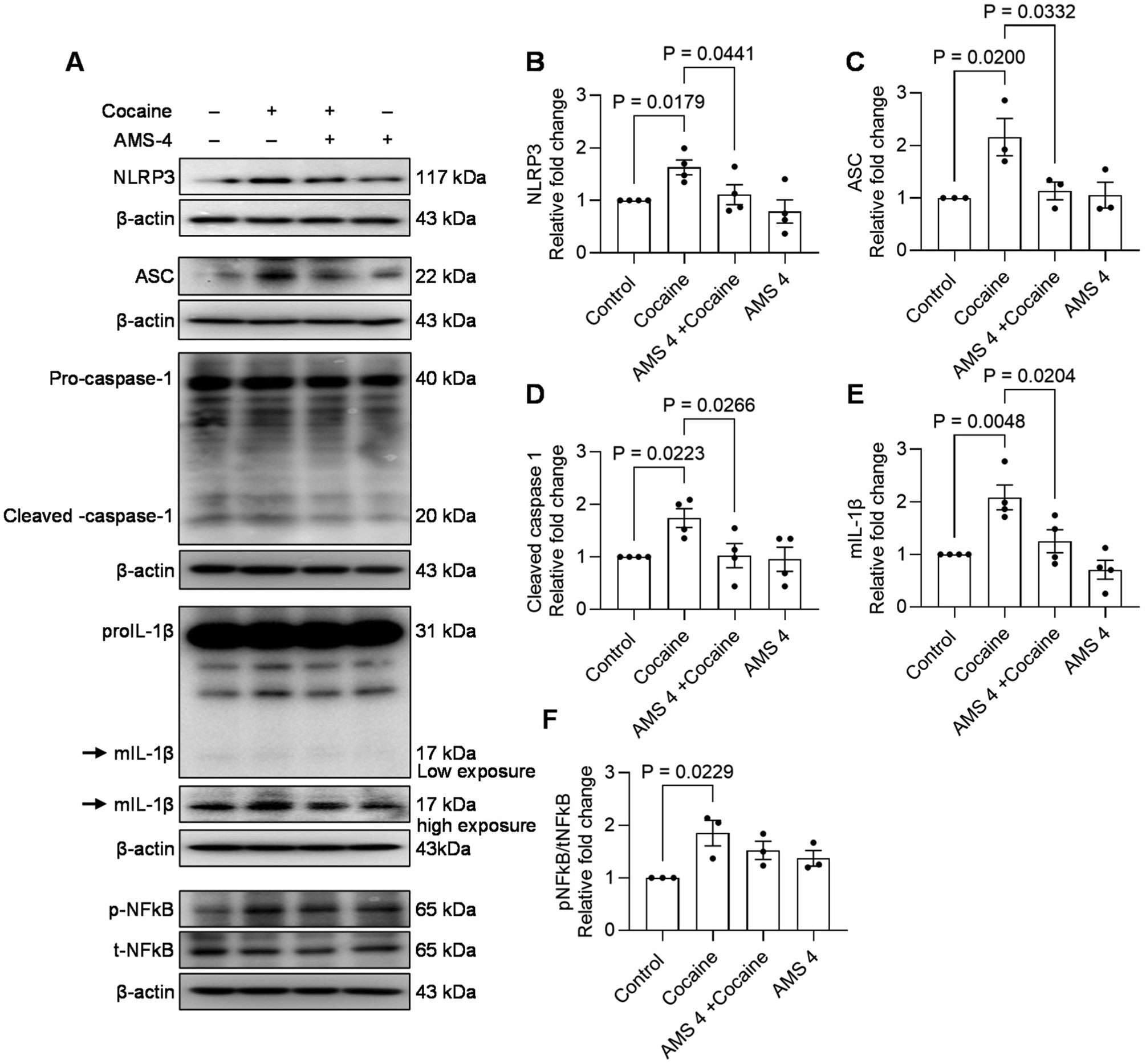
AMS-4 selectively attenuates cocaine-induced NLRP3 inflammasome activation in BV2 microglial cells. (A) Representative western blots showing the protein expression of NLRP3, ASC, cleaved caspase-1, mature IL-1β, and phospho-NFκB in BV2 cells pretreated with AMS-4 (25 μM, 1 h) followed by cocaine exposure (10 μM, 24 h). β-Actin was used as a loading control. (B–F) Densitometric quantification of NLRP3, ASC, cleaved caspase-1, mature IL-1β and phospho-NFκB protein levels normalized to β-actin. Data are presented as mean ± SEM; N = 3-4.

Based on the ability of AMS-4 to suppress the activation phase (signal 2) of NLRP3 inflammasome signaling, we next examined its effects on inflammasome assembly and downstream effector activation in cocaine-exposed BV2 microglial cells. BV2 cells were pretreated with AMS-4 (25 μM) for 1 h prior to cocaine exposure (10 μM). AMS-4 significantly reduced cocaine-induced expression of NLRP3 and ASC, the key components required for inflammasome assembly (Fig. 4A-C). Consistent with impaired inflammasome activation, AMS-4 markedly attenuated cocaine-induced caspase-1 activation (Fig. 4A and D) and reduced mature IL-1β production (Fig. 4A and E). In contrast to AMS-17, however, AMS-4 pretreatment did not alter cocaine-induced NFκB expression (Fig. 4A and F), indicating that AMS-4 does not affect the priming phase (signal 1) of NLRP3 inflammasome activation. Collectively, these results demonstrate that AMS-4 selectively inhibits cocaine-induced NLRP3 inflammasome activation at the assembly and activation stage without interfering with upstream NFκB-dependent priming.

## 4. Discussion

Building on the structure of the natural product isoliquiritigenin, we previously designed the NLRP3 inflammasome inhibitor AMS-17 [18]. The design strategy focused on two key modifications: (1) replacing the electron-deficient enone moiety with a sulfonylurea group to enhance drug likeness, and (2) substituting the phenolic groups with metabolically stable alternatives to improve pharmacokinetic properties. In our earlier studies, AMS-17 significantly reduced levels of IL-1β, inducible nitric oxide synthase (iNOS), and TNF-α in N9 microglial cells [18]. More recently, we demonstrated its neuroprotective effects of AMS-17 *in vivo* using a rodent model of vascular dementia [20]. Based on these findings, we hypothesized that AMS-17 and its structural analogues would exert similar neuroprotective effects in the context of CUD via inhibiting the NLRP3 inflammasome. In this study, we aimed to briefly investigate the structure-activity relationship (SAR) of the two aromatic ring systems in AMS-17. Analogue AMS-4 features substitution of the pyrimidine ring in AMS-17 with an indole moiety, while AMS-6 incorporates a benzoxazole ring in place of the pyrimidine ring. Additionally, the substituted phenyl ring of AMS-17 was replaced with a pyridine ring in AMS-7 and a thiophene ring in AMS-8 to further probe the impact of heteroaromatic ring systems on biological activity.

To evaluate the neuroprotective potential of AMS-17 and its structurally modified analogues, we assessed their ability to inhibit cocaine-induced NLRP3 activation in BV2 microglial cells. Our findings demonstrate that multiple AMS analogues (AMS-4, AMS-6, and AMS-8) suppressed NLRP3 expressions in BV2 microglial cells exposed to cocaine, thus suggesting an anti-inflammatory potential of this series of tertiary sulfonylurea compounds. In contrast, AMS-7 failed to produce an appreciable reduction in NLRP3 expression, even at higher concentrations. Among the analogues tested, AMS-4 exhibited the most potent inhibitory effect on NLRP3 expression, highlighting its efficacy in modulating cocaine mediated microglial inflammasome activation.

The current study focused on AMS-4 and its parent compound AMS-17, for their modulation in cocaine mediated NLRP3 inflammasome activation. Both AMS-4 and AMS-17 were significantly able to reduce the ASC oligomerization that in turn was able to reduce the caspase-1 cleavage and eventually the mature IL-1β, a hallmark of inflammasome activation. The results suggested that AMS-4 effectively inhibited the activation phase of the NLRP3 inflammasome cascade. While the analogues were effective in inhibiting the NLRP3 signaling, further studies pertaining to stability, off-target effects and blood brain penetrance are warranted to assess the effectiveness and safety of these molecules in the *in vivo* setting. These findings highlight the critical role of the two aromatic rings in the biological activity of sulfonylurea compounds. Sulfonylurea compounds derived from aminopyrimidine (AMS-17), amino-indole (AMS-4), and amino-benzoxazole (AMS-6) displayed good biological activity. Sulfonylureas derived from 4-trifluoromethyl-substituted phenyl ring (AMS-17) show good activity. Replacement of 4-substituted phenyl ring with pyridine (AMS-7) or thiophene (AMS-8) rings resulted in significant loss of biological activity.

Overall, these findings position sulfonylurea compounds, such as AMS-4 as a promising lead compound for the suppression of microglial NLRP3 inflammasome activity in cocaine-induced neuroinflammation. Further studies in primary microglia and *in vivo* models are warranted to elucidate its molecular targets and therapeutic potential. Continued exploration of the structure–activity relationships among AMS analogues will aid future optimization efforts for anti-neuroinflammatory drug development.
